# The UK BiLEVE and Mendelian randomisation: using multivariable instrumental variables to address “damned if you, damned if you don’t” adjustment problems

**DOI:** 10.1186/s13104-023-06434-8

**Published:** 2023-07-25

**Authors:** Benjamin Woolf, Dipender Gill, Hannah Sallis, Marcus R. Munafò

**Affiliations:** 1 School of Psychological Science, University of Bristol, Bristol, UK; 2 MRC Integrative Epidemiology Unit, University of Bristol, Bristol, UK; 3 MRC Biostatistics Unit, University of Cambridge, Cambridge, UK; 4 Research and Early Development, Novo Nordisk, Copenhagen, Denmark; 5 Department of Epidemiology and Biostatistics, School of Public Health, Imperial College London, London, UK; 6 Centre for Academic Mental Health, Population Health Sciences, Bristol Medical School, University of Bristol, Bristol, UK

## Abstract

**Objective:**

To explore the use of multivariable instrumental variables to resolve the “damned if you do, damned if you don’t” adjustment problem created for Mendelian randomisation (MR) analysis using the smoking or lung function related phenotypes in the UK Biobank (UKB).

**Result:**

“damned if you do, damned if you don’t” adjustment problems occur when both adjusting and not-adjusting for a variable will induce bias in an analysis. One instance of this occurs because the genotyping chip of UKB participants differed based on lung function/smoking status. In simulations, we show that multivariable instrumental variables analyses can attenuate potential collider bias introduced by adjusting for a proposed covariate, such as the UKB genotyping chip. We then explore the effect of adjusting for genotyping chip in a multivariable MR model exploring the effect of smoking on seven medical outcomes (lung cancer, emphysema, hypertension, stroke, heart diseases, depression, and disabilities). We additionally compare our results to a traditional univariate MR analysis using genome-wide analyses summary statistics which had and had not adjusted for genotyping chip. This analysis implies that the difference in genotyping chip has introduced only a small amount of bias.

**Supplementary Information:**

The online version contains supplementary material available at 10.1186/s13104-023-06434-8.

## Introduction

Mendelian randomisation (MR) is an increasingly popular method for inferring the causal effect of modifiable exposures on epidemiological outcomes [1]. In an MR study, genetic variants which are robustly associated with the exposure of interest are used as instruments in an instrumental variables analysis.

One of the most popular resources for conducting MR analyses is the UK Biobank (UKB) [2]. The UKB is a large (approximately half a million participants) population cohort study of Britons. The UKB has been used in the MR literature to explore the causal effects of smoking-related phenotypes [3]. However, the UKB genotyping was rolled out over several steps.

Participants enrolled in the UK BiLEVE study were genotyped using different instruments (‘genotyping chip’) than other participants [4]. Because UK BiLEVE was not randomly sampled, there is a worry that differences in genotyping between participants could cause (confounding) bias if the sampling probability is associated with risk factors for the outcome phenotype of interest. Because of this, there is general advice to adjust UKB GWASs by genotyping chip [5]. However, the UK BiLEVE study selected participants who were in a tail or centre of the distribution for lung function and smoking. This poses a problem for genetic designs, like MR, in the UKB exploring smoking and lung function-related phenotypes. If genotyping chip is determined by UK BiLEVE enrolment, which is in turn determined by lung function and smoking status, then adjusting for genotyping chip could introduce collider bias.

**Fig. 1:**
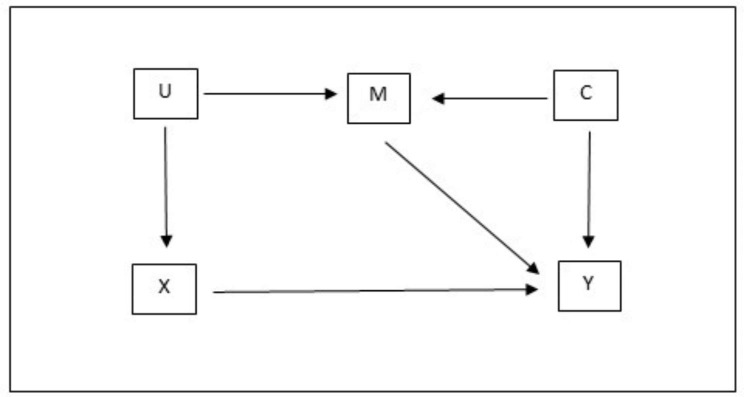
An example “damned if you do, damned if you don’t” adjustment problem. Adjusting for M will result in collider bias, but not adjusting for M will introduce confounding

Situations where no adjustment will result in bias, but where the required covariate is a collider (see Fig. 1) have been described as “damned if you do, damned if you don’t” adjustment problems in a recent review of types of covariate controls [6]. This review concluded that there were no satisfactory methods for addressing this bias, and they suggested authors should implement sensitivity analyses when encountering this type of problem. Existing guidelines for conducting genetic analysis in the UKB had made similar suggestions of running analyses with and without adjustment for genotyping chip when a phenotype might relate to lung function or smoking [5].

In this article, we propose a complementary sensitivity analysis to address this type of adjustment paradox, with specific emphasis on addressing bias due to UK BiLEVE in MR studies. Multivariable IV (MVIV) is an extension of traditional IV analysis to include more than one exposure [7]. A traditional IV analysis, like MR, assumes that the instrument is robustly associated with the exposure, can affect the outcome only via the exposure, and that there is no ‘back door’ path from the instrument to the outcome. MVIV modifies these assumptions so that: 1) the instrument is robustly associated with the exposure(s) *conditional on the other covariate(s)*, can affect the outcome only via *one of* the exposures, and that, *conditional on all covariates*, there is no ‘back door’ path from the instrument to the outcome. While the effect estimates of a standard IV analysis are the total effect of the exposure on the outcome, MVIV effect estimates should be interpreted as the direct effect of the exposure conditional on the covariates. Because of this, MR applications of MVIV have shown that it can be used to address bias, like collider bias [8, 9], by ensuring that the effect estimate of interest is conditionally independent of a known biasing phenotype. Intuitively then, adding the genotyping chip as a second exposure to an MR model using chip adjusted genome-wide summary statistics should remove any collider bias introduced by adjusting for genotyping chip. The “damned if you do, damned if you don’t” paradoxes explored here differs from existing applications of MVIV which have focused on settings where the variable being adjusted for is only one of a confounder [10], pleiotropic [7], or a collider [8, 9], whereas here we consider a setting where it is both a collider and either pleiotropic or a confounder.

**Fig. 2:**
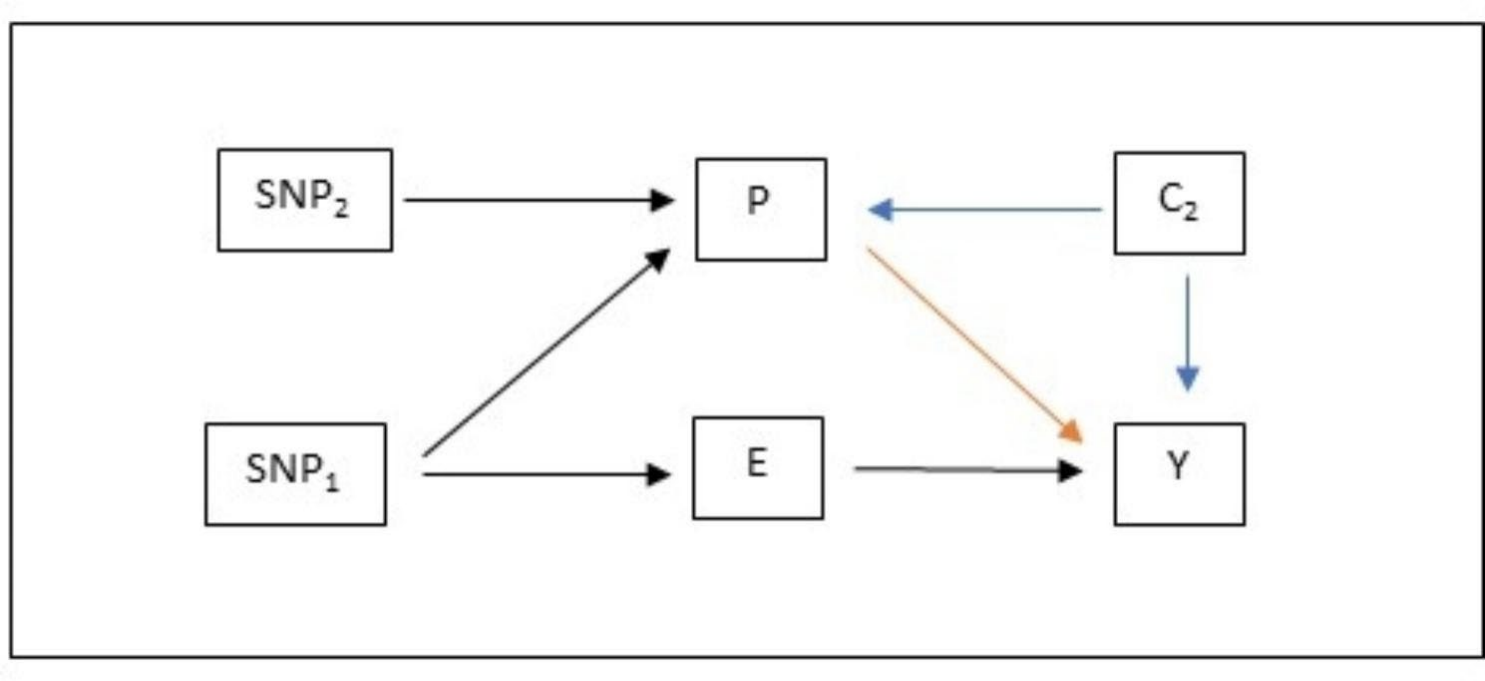
Directed Acyclic Graph of data generative model used in simulation. Here, G1 is the genetic liability to the exposure, and G2 the genetic liability to the potential covariate. C is the potential covariate, and U1 and U2 are confounders. X and Y are the exposure and outcome respectively. M is additionally pleotropic and a collider

## Main text

### Simulation

#### Aims

We ran a simple simulation to provide a proof of concept that MVIV can be used to address “damned if you do, damned if you don’t” adjustment problems. We report our simulations using the ADEMP (aims, data-generating mechanisms, estimands, methods, and performance measures) approach [11].

#### Data-generating mechanisms

We simulated a setting in which there is an exclusion restriction violation (i.e. where the instrument causes the outcome via a path not mediated by the exposure), but where adjusting for this violation would introduce M bias (Fig. 2). More formally, we simulated 100 single nucleotide polymorphisms (SNPs, which are common genetic variants) as independent and identically distributed binomial variables with the following parameters.

$$SNP \sim B\left( {2,N\left( {0.5,{{0.1}^2}} \right)} \right)$$

The distribution of allele frequencies was chosen to be loosely based on the one observed for the genome wide significant SNPs in the UKB GWAS of lifetime smoking by Wooton and colleagues [3].

We then simulated two confounders as independent and identically distributed normal variables with the following parameters:

$$C \sim N\left( {0,{1^2}} \right)$$

We then defined the exposure as.

$$E = {C_1} + \sum\nolimits_1^{50} {\left[ {N\left( {0.1,{{0.05}^2}} \right) * SNP} \right]} + {\varepsilon _1}$$

where ε is an error term such that ε_1_ ~ N(0, 1^2^).

We defined the potential covariate for blocking the exclusion restriction violation as.

$$P = {C_2} + \sum\nolimits_{51}^{100} {\left[ {N\left( {0.1,{{0.05}^2}} \right) * SNP} \right]} + {\varepsilon _2}$$

where ε_2_ ~ N(0, 1^2^).

Finally we defined the outcome as.

$$Y = E + {C_1} + {C_2} + P + {\varepsilon _3}$$

where ε_3_ ~ N(0, 1^2^).

The phenotypic beta values chosen in this simulation were chosen purely arbitrarily. However, biases are generally more visible with larger effect estimates. By choosing betas values of 1 we therefore hoped to clearly illustrate any possible effect of using MVIV. The exact conclusions of our simulation are therefore not expected to generalise to any specific applied setting.

#### Estimands

The causal effect of the exposure on the outcome.

Methods: We compare three methods for estimating the causal effect of the exposure on the outcome:

1. We ran an inverse variance weighted (intercept free) regression of B_y_ ~ B_x_ + 0, where B_y_ is the SNP-outcome association and B_x_ is the SNP-exposure association, and where the B_x_ and B_y_ were estimated in linear models which additionally adjusted for P.
2. An inverse-variance weighted (intercept free) regression of B_y_ ~ B_x_ + 0, where the B_x_ and B_y_ were not estimated in linear models which did not additionally adjust for P.
3. An inverse-variance weighted (intercept free) regression of B_y_ ~ B_x_ + B_p_ + 0, where B_p_ is the SNP-covariate association, and where the B_x_ and B_y_ were estimated in linear models which additionally adjusted for P.

For readers less familiar with the MR literature, it is worth noting that the intercept-free weighted regression is equivalent to an inverse variance weighted meta-analysis of the IV Wald ratios for each SNP. In addition, [1] and [2] only used the first 50 simulated SNPs (i.e. those which associated with the exposure), while [3] used all 100 simulated SNPs (i.e. SNPs which associated with either exposure). B_y_, B_x_, and B_p_ were all estimated in non-overlapping samples of 250,000 participants.

#### Performance measure

The mean bias in the causal effect of the exposure on the outcome over 1000 iterations.

#### Results of the simulation

The simulation found that both adjusting and not adjusting the linear model for the covariate resulted in bias (mean bias = -0.445 [MC SE = 0.002] and 0.972 [MC SE = 0.001] for the adjusted and not adjusted analysis respectively). On the other hand, the MVIV model attenuated most of the bias (mean bias = -0.064 [MC SE = 0.002]).

### Applied example with the UKB genotyping chip

We used a two-sample MR analysis of the effect of smoking on seven outcomes (lung cancer, emphysema, depression, hypertension, stroke, heart diseases, and diabetes) in the UKB as an applied example. The outcomes were chosen because there is existing literature implying a causal association between smoking and these outcomes [12–17]. We ran three versions of this analysis: (1) using univariable MR to estimate the effect of smoking on the outcomes where the UKB smoking, lung cancer and emphysema GWASs had adjusted for genotyping chip, (2) using univariable MR to estimate the effect of smoking on the outcomes where the UKB smoking, lung cancer and emphysema GWASs had not adjusted for genotyping chip, and (3) using multivariable MR estimate the effect smoking on the outcomes adjusted for genotyping chip where the UKB smoking, lung cancer and emphysema GWASs had adjusted for genotyping chip.

We used the Wootton et al. UKB lifetime smoking GWAS as a source of SNP-exposure associations [3], which we standardised by dividing the effect estimate and standard error by 0.6940093. To estimate genotype-chip association we ran a GWAS (adjusted for age, sex, and the first 10 principal components of ancestry) using BOLT-LMM in the MRC-IEU UKB GWAS pipeline. Full methods for both GWASs described elsewhere [3, 18]. In both the univariate and multivariate setting, we selected genome-wide significant SNPs associated with the exposure(s) of interest as genetic instruments, and then clumped this list using an r^2^ of 0.001 and kb of 10,000. We additionally implemented the FIQT WCC on the exposure GWASs to correct for any effect of Winner’s curse [19].

We used the Elsworth’s UKB GWASs in the MRC-IEU OpenGWAS platform as a source of SNP-emphysema and -hypertension associations [20]; as well as Nikpay et al’s GWAS of CAD, Malik et al’s GWAS of stroke, Wang et al’s GWAS of lung cancer, Howard et al’s GWAS of depression and the FinnGen round 5 GWAS of disabilities [21–25]. Details on genotyping, quality control, and phenotyping can be found in the original publications and on the UKB website (https://biobank.ndph.ox.ac.uk/ukb/search.cgi). All outcome GWASs were on the odds ratio scale. We harmonised the exposure and outcome samples, and removed palindromic SNPs whose effect allele could not be inferred using based on minor allele frequency. We used four MR estimators: IVW, weighted mode, weighted median, and MR Egger. We additionally estimated the heterogeneity in the MR Wald ratios using the Cochrane Q statistic as a control for exclusion restriction violations. The univariate MR analysis was implemented using the TwoSampleMR R package [26, 27]. Multivariable MR analyse additionally used the MVMR, MendelianRandomization, and MVMRMode R packages [28–30].

Table 1 presents the results of this analysis. These broadly show highly consistent findings across the three methods, with most of the changes in estimates smaller than the standard error of each point estimate. Overall, this would therefore broadly imply that there is minimal collider or confounding bias introduced by adjusting or not adjusting for genotyping chip.

## Limitations

Here we have shown that MVIV can, in theory, be used to attenuate bias when “damned if you do, damned if you don’t” adjustment problems occur in an IV analysis. We then apply this to the UKB and show that, despite differences in genotyping depending on lung function and smoking status of participants, the UK BiLEVE study appears to have introduced only a small amount of bias into our estimates of the causal effect of smoking on lung cancer.

There are three complications to the application of our findings to address differences in genotyping chip in the UKB, which we believe mean that our proposal should be used to supplement, rather than replace the existing guidance of performing both a chip-adjusted, and no-chip-adjusted, analysis as a sensitivity analysis. Firstly, MVIV has additional parameters than univariable IV analyses, and will therefore be even less precise. Secondly, the collinearity of exposures (such as smoking and genotype chip) can also introduce conditionally weak instruments into analyses which would have strong instruments in a university setting. Although not an issue in our applied analysis, this could become a major issue in analysis using weaker instruments, such as parental smoking status. Hence, authors should come to a judgment about which method will have a lower mean squared error and then use the alternatives as sensitivity analyses. Thirdly, because enrolment into the UK BiLEVE study was determined by smoking and lung function, it could be argued that it is, in effect, a proxy of these variables. If this is the case, then adjusting for genotyping chip in a model would potentially do something equivalent to adjusting for a mediator in a traditional regression analysis, and therefore introduce bias. This underpins the importance of not using the MVIV analysis to replace the existing guidelines.

A final, but related, limitation when applying our proposal to other settings is that there has to be a way to validly instrument the proposed covariate. Since there are many settings, especially when using summary data IV analysis like two-sample MR, when study-specific variables, such as UKB genotyping chip, this may be more common than the authors would hope.

**Table 1:** Results of applied example. UVMR = univariable Mendelian randomisation, MVMR = multivariable Mendelian randomisation

Phenotype	Analysis	IVW	MR Egger	Weighted Median	Weighted Mode	Number of SNPs	F statistic: phenotype (chip)	Cochrane Q
Diabetes	UVMR, no chip adjustment	0.314 (0.150 to 0.477)	-0.086 (-0.761 to 0.590)	0.258 (0.041 to 0.474)	0.231 (-0.217 to 0.680)	124	41.917	0.000
	UVMR, chip adjustment	0.240 (0.061 to 0.418)	-0.018 (-0.784 to 0.748)	0.242 (0.019 to 0.465)	0.203 (-0.232 to 0.638)	118	42.139	0.000
	MVMR, chip adjustment	0.302 (0.121 to 0.483)	-0.076 (-0.596 to 0.443)	0.259 (0.026 to 0.493)	0.311 (0.135 to 0.471)	215	351.575 (193.090)	0.000
Lung cancer	UVMR, no chip adjustment	1.454 (1.121 to 1.788)	3.336 (1.997 to 4.675)	1.084 (0.703 to 1.466)	0.968 (-0.011 to 1.948)	122	42.166	0.000
	UVMR, chip adjustment	1.463 (1.098 to 1.827)	3.041 (1.483 to 4.600)	1.016 (0.619 to 1.413)	0.767 (-0.359 to 1.892)	116	42.431	0.000
	MVMR, chip adjustment	1.352 (1.006 to 1.699)	1.930 (0.868 to 2.993)	0.994 (0.557 to 1.431)	1.236 (0.808 to 1.571)	178	413.201 (163.361)	0.000
Chronic bronchitis/ emphysema	Univariable, no chip adjustment	0.229 (0.194 to 0.264)	0.339 (0.212 to 0.466)	0.210 (0.161 to 0.258)	0.189 (0.055 to 0.322)	132	42.370	0.003
	Univariable, chip adjustment	0.247 (0.206 to 0.288)	0.426 (0.256 to 0.597)	0.242 (0.190 to 0.294)	0.262 (0.109 to 0.415)	117	42.280	0.001
	MVMR, chip adjustment	0.237 (0.190 to 0.285)	0.346 (0.204 to 0.488)	0.220 (0.157 to 0.284)	0.299 (0.230 to 0.369)	120	377.066 (54.873)	0.004
Depression	UVMR, no chip adjustment	0.485 (0.379 to 0.5910	-0.009 (-0.404 to 0.385)	0.394 (0.289 to 0.500)	0.282 (-0.001 to 0.565)	121	42.196	0.000
	UVMR, chip adjustment	0.473 (0.353 to 0.594)	0.159 (-0.338 to 0.656)	0.469 (0.358 to 0.581)	0.459 (0.163 to 0.755)	116	41.872	0.000
	MVMR, chip adjustment	0.558 (0.454 to 0.662)	0.293 (0.012 to 0.575)	0.534 (0.411 to 0.658)	0.389 (0.282 to 0.563)	201	367.184 (177.232)	0.000
Heart disease	UVMR, no chip adjustment	0.449 (0.295 to 0.603)	-0.088 (-0.717 to 0.541)	0.423 (0.219 to 0.627)	0.155 (-0.426 to 0.735)	125	41.890	0.000
	UVMR, chip adjustment	0.453 (0.288 to 0.617)	-0.371 (-1.061 to 0.320)	0.522 (0.313 to 0.731)	0.617 (-0.021 to 1.254)	119	42.191	0.000
	MVMR, chip adjustment	0.423 (0.248 to 0.598)	-0.295 (-0.820 to 0.229)	0.445 (0.209 to 0.682)	0.490 (0.332 to 0.652)	201	373.412 (183.898)	0.000
High blood pressure	UVMR, no chip adjustment	0.319 (0.187 to 0.451)	-0.050 (-0.552 to 0.453)	0.202 (0.078 to 0.325)	0.214 (-0.011 to 0.439)	124	41.997	0.000
	UVMR, chip adjustment	0.264 (0.132 to 0.396)	-0.056 (-0.587 to 0.474)	0.234 (0.121 to 0.347)	0.274 (0.044 to 0.505)	120	42.139	0.000
	MVMR, chip adjustment	0.309 (0.195 to 0.423)	0.001 (-0.304 to 0.305)	0.291 (0.170 to 0.412)	0.347 (0.244 to 0.450)	216	349.781 (192.446)	0.000
Stroke	UVMR, no chip adjustment	0.307 (0.193 to 0.421)	-0.053 (-0.515 to 0.408)	0.270 (0.106 to 0.434)	0.233 (-0.096 to 0.561)	123	41.956	0.230
	UVMR, chip adjustment	0.243 (0.127 to 0.360)	-0.119 (-0.623 to 0.385)	0.207 (0.032 to 0.381)	0.153 (-0.261 to 0.568)	118	42.227	0.347
	MVMR, chip adjustment	0.265 (0.128 to 0.402)	-0.035 (-0.462 to 0.393)	0.276 (0.071 to 0.480)	0.244 (0.096 to 0.393)	180	416.145 (169.530)	0.061

## Electronic supplementary material

Below is the link to the electronic supplementary material.

Supplementary Material 1

## Data Availability

The code and GWAS summary statistics used in this study is available from 10.17605/OSF.IO/RPBYD. The GWAS summary statistics will also be made available from the MRC-IEU OpenGWAS project. This project was conducted using UK Biobank application no. 15,825. UK Biobank was established by the Wellcome Trust medical charity, Medical Research Council, Department of Health, Scottish Government and the Northwest Regional Development Agency. It has also had funding from the Welsh Government, British Heart Foundation, Cancer Research UK and Diabetes UK. UK Biobank is supported by the National Health Service (NHS). UK Biobank is open to bona fide researchers anywhere in the world.
