# Supplementary material for: The UK BiLEVE and Mendelian randomisation: using multivariable instrumental variables to address “damned if you, damned if you don’t” adjustment problems
Source: BMC Res Notes. 2023 Jul 25;16:157. doi: 10.1186/s13104-023-06434-8 (PMC10369748; doi:10.1186/s13104-023-06434-8)
Supplement: Supplementary file 1 — Supplementary Material 1 [file 13104_2023_6434_MOESM1_ESM.docx]

**Supplementary Table 1: Applied MR results**

| Description | IVW | IVW_LCI | IVW_UCI | egger | egger_lci | egger_uci | median | median_lci | median_uci | mode | mode_LCI | mode_UCI | nsnp | F_1 | F_2 | I2_1 | I2_2 | Q_p | Phenotype |
| --- | --- | --- | --- | --- | --- | --- | --- | --- | --- | --- | --- | --- | --- | --- | --- | --- | --- | --- | --- |
| Univar, no chip adjustment | 0.314 | 0.15 | 0.477 | -0.086 | -0.761 | 0.59 | 0.258 | 0.041 | 0.474 | 0.231 | -0.217 | 0.68 | 124 | 41.917 | NA | 0.976 | NA | 0 | Diabetes |
| Univar, chip adjustment | 0.24 | 0.061 | 0.418 | -0.018 | -0.784 | 0.748 | 0.242 | 0.019 | 0.465 | 0.203 | -0.232 | 0.638 | 118 | 42.139 | NA | 0.976 | NA | 0 | Diabetes |
| MVMR, chip adjustment | 0.302 | 0.121 | 0.483 | -0.076 | -0.596 | 0.443 | 0.259 | 0.026 | 0.493 | 0.311 | 0.135 | 0.471 | 215 | 351.575 | 193.09 | 0.952 | 0.944 | 0 | Diabetes |
| Univar, no chip adjustment | 1.454 | 1.121 | 1.788 | 3.336 | 1.997 | 4.675 | 1.084 | 0.703 | 1.466 | 0.968 | -0.011 | 1.948 | 122 | 42.166 | NA | 0.976 | NA | 0 | Lung cancer |
| Univar, chip adjustment | 1.463 | 1.098 | 1.827 | 3.041 | 1.483 | 4.6 | 1.016 | 0.619 | 1.413 | 0.767 | -0.359 | 1.892 | 116 | 42.431 | NA | 0.977 | NA | 0 | Lung cancer |
| MVMR, chip adjustment | 1.352 | 1.006 | 1.699 | 1.93 | 0.868 | 2.993 | 0.994 | 0.557 | 1.431 | 1.236 | 0.808 | 1.571 | 178 | 413.201 | 163.361 | 0.959 | 0.934 | 0 | Lung cancer |
| Univar, no chip adjustment | 0.229 | 0.194 | 0.264 | 0.339 | 0.212 | 0.466 | 0.21 | 0.161 | 0.258 | 0.189 | 0.055 | 0.322 | 132 | 42.37 | NA | 0.977 | NA | 0.003 | Chronic bronchitis/emphysema |
| Univar, chip adjustment | 0.247 | 0.206 | 0.288 | 0.426 | 0.256 | 0.597 | 0.242 | 0.19 | 0.294 | 0.262 | 0.109 | 0.415 | 117 | 42.28 | NA | 0.977 | NA | 0.001 | Chronic bronchitis/emphysema |
| MVMR, chip adjustment | 0.237 | 0.19 | 0.285 | 0.346 | 0.204 | 0.488 | 0.22 | 0.157 | 0.284 | 0.299 | 0.23 | 0.369 | 120 | 377.066 | 54.873 | 0.973 | 0.838 | 0.004 | Chronic bronchitis/emphysema |
| Univar, no chip adjustment | 0.485 | 0.379 | 0.591 | -0.009 | -0.404 | 0.385 | 0.394 | 0.289 | 0.5 | 0.282 | -0.001 | 0.565 | 121 | 42.196 | NA | 0.976 | NA | 0 | Depression |
| Univar, chip adjustment | 0.473 | 0.353 | 0.594 | 0.159 | -0.338 | 0.656 | 0.469 | 0.358 | 0.581 | 0.459 | 0.163 | 0.755 | 116 | 41.872 | NA | 0.976 | NA | 0 | Depression |
| MVMR, chip adjustment | 0.558 | 0.454 | 0.662 | 0.293 | 0.012 | 0.575 | 0.534 | 0.411 | 0.658 | 0.389 | 0.282 | 0.563 | 201 | 367.184 | 177.232 | 0.954 | 0.94 | 0 | Depression |
| Univar, no chip adjustment | 0.449 | 0.295 | 0.603 | -0.088 | -0.717 | 0.541 | 0.423 | 0.219 | 0.627 | 0.155 | -0.426 | 0.735 | 125 | 41.89 | NA | 0.976 | NA | 0 | Heart disease |
| Univar, chip adjustment | 0.453 | 0.288 | 0.617 | -0.371 | -1.061 | 0.32 | 0.522 | 0.313 | 0.731 | 0.617 | -0.021 | 1.254 | 119 | 42.191 | NA | 0.976 | NA | 0 | Heart disease |
| MVMR, chip adjustment | 0.423 | 0.248 | 0.598 | -0.295 | -0.82 | 0.229 | 0.445 | 0.209 | 0.682 | 0.49 | 0.332 | 0.652 | 201 | 373.412 | 183.898 | 0.955 | 0.94 | 0 | Heart disease |
| Univar, no chip adjustment | 0.319 | 0.187 | 0.451 | -0.05 | -0.552 | 0.453 | 0.202 | 0.078 | 0.325 | 0.214 | -0.011 | 0.439 | 124 | 41.997 | NA | 0.976 | NA | 0 | High blood pressure |
| Univar, chip adjustment | 0.264 | 0.132 | 0.396 | -0.056 | -0.587 | 0.474 | 0.234 | 0.121 | 0.347 | 0.274 | 0.044 | 0.505 | 120 | 42.139 | NA | 0.976 | NA | 0 | High blood pressure |
| MVMR, chip adjustment | 0.309 | 0.195 | 0.423 | 0.001 | -0.304 | 0.305 | 0.291 | 0.17 | 0.412 | 0.347 | 0.244 | 0.45 | 216 | 349.781 | 192.446 | 0.952 | 0.944 | 0 | High blood pressure |
| Univar, no chip adjustment | 0.307 | 0.193 | 0.421 | -0.053 | -0.515 | 0.408 | 0.27 | 0.106 | 0.434 | 0.233 | -0.096 | 0.561 | 123 | 41.956 | NA | 0.976 | NA | 0.23 | Stroke |
| Univar, chip adjustment | 0.243 | 0.127 | 0.36 | -0.119 | -0.623 | 0.385 | 0.207 | 0.032 | 0.381 | 0.153 | -0.261 | 0.568 | 118 | 42.227 | NA | 0.976 | NA | 0.347 | Stroke |
| MVMR, chip adjustment | 0.265 | 0.128 | 0.402 | -0.035 | -0.462 | 0.393 | 0.276 | 0.071 | 0.48 | 0.244 | 0.096 | 0.393 | 180 | 416.145 | 169.53 | 0.959 | 0.934 | 0.061 | Stroke |

Key: description = description of analysis, IVW = beta of IVW effect, IVW_LCI = lower 95% confidence interval of IVW effect, IVW_UCI = upper 95% confidence interval of IVW effect, egger = MR Egger effect, egger_lci = lower 95% confidence interval of MR egger effect, egger_uci = upper 95% confidence interval of MR Egger effect, median = Weighted median effect, median_lci = lower 95% confidence interval of Weighted median effect, median_uci = upper 95% confidence interval of Weighted median effect, Mode = Weighted mode effect, mode_LCI = lower 95% confidence interval of Weighted mode effect, mode_UCI uci = upper 95% confidence interval of Weighted mode effect, nsnp = number of SNPs, F_1 = F statistic of smoking, F_2 = F statstic of genotyping chip, I2_1 = I^2 statistics of smoking, I2_2 = I^2 statstic of genotyping chip, Q_p = Cochrane Q stat of SNP effects, phenotype = outcome phenotype.
